# Evaluation of growth parameters and body condition score on weaning stages of Saanen goats

**DOI:** 10.5455/javar.2022.i622

**Published:** 2022-09-30

**Authors:** Noor Syaheera Ibrahim, Nurin Nabila Mohd Noor, Nur Najwa Asyikin Binti Muhammad Nasruddin

**Affiliations:** School of Animal Science, Aquatic Science, and Environment, Faculty of Bioresources and Food Industry, Universiti Sultan Zainal Abidin, Kuala Terengganu, Malaysia

**Keywords:** Growth parameters, body condition score, weaning stages, Saanen goat

## Abstract

**Objective:**

There are three objectives in this study; 1) to measure the live weight of pre-and post-weaning Saanen kids; 2) to determine the growth curves between males and females pre-and post-weaning Saanen kids; and 3) to analyze the body condition score (BCS) between male and female pre-and post-weaning kids.

**Materials and Methods:**

This study included pre-weaning kids aged 1 month, as well as post-weaning kids aged 3–6 months. 10 pre-weaning purebred Saanen kids (*n* = 5 male and *n* = 5 female) and 20 post-weaning Saanen kids (*n* = 10 male and *n* = 10 female) were employed in this investigation. Pre-and post-weaning kids’ live weights were assessed weekly on a weighing scale, and BCS was calculated based on their body frame. In the data analysis, the two-sample t-test with Minitab Software was utilized.

**Results:**

The findings revealed that pre-weaning Saanen kids gained weight steadily from week 1 to week 6, with males being heavier than females. The p-value, on the other hand, suggested that there was no difference in live weight between pre-weaning male and female Saanen kids. Over 6 weeks of sampling, the male had a larger proportion of live weight gain (80%) than the female (75%). Meanwhile, the BCS of pre-weaning Saanen kids grew from week 1 to week 6. It is critical to account for the development of muscle mass while still evaluating the fat cover to determine whether the kids are maintaining an adequate BCS. However, the live weight of post-weaning kids was inconsequential because they were still in the growing phase. As a result, from the 1st to 6th week, post-weaning kids’ body weight and BCS increased as their growth progressed. After 6 weeks of sampling, females had a higher percentage of live weight than males. This is because the kids raised on the farm do not have complete control over the environmental effects. Over 6 weeks of sampling, female post-weaning Saanen kids grew a slightly higher percentage of live weight (88%) than males (85%).

**Conclusion:**

This study conducted a direct assessment study, which monitored and determined the live weight and BCS of pre-and post-weaning Saanen kids. Pre-weaning kids’ average values of live weight were calculated as insignificant at the age of 1 month. The mean live weight is most affected by milk consumption from its mothers, the management of the farm, and the environment. For the post-weaning Saanen kids, the females have a slightly higher average live weight gained for 6 weeks than the males (*p* > 0.05). In conclusion, the live weight changes of Saanen kids during the weaning stages are independent of the BCS.

## Introduction

Commercial dairy goat farms have been established in numerous Southeast Asian countries over the past couple of decades because of the rising consumer demand for goat milk [1]. Smallholders are starting to open more dairy goat farms in Malaysia because of the rising demand for animal products. However, this is insufficient to meet demand, and Malaysia must import goat products, mainly milk and meat, from other countries [2]. One of the breeds of dairy goats with the largest bodies is the Saanen goat, which may mature at a live weight of up to 55 kg and a height of up to 80 cm [3]. Saanen goats are also referred to as dual-purpose breeds because they can produce both milk and meat. Goat milk is well-liked in Malaysian society because of its medicinal benefits and religious importance [4,5]. Goat farming is one of the important industries in the agro-food subsector in Malaysia [6]. Goats provide a source of meat and milk for Malaysians’ nutritional requirements. Currently, 29.5% of registered livestock farms in west Malaysia are devoted to small ruminant populations [7]. Goat milk is essential to produce cheese and yogurt instead of milk and meat. Weaning young animals should therefore take the place of breeding stock whenever possible because doing so increases farm output and satisfies the objectives of the dairy sector, provided that the animals can live and grow unharmed [8,9].

Saanen kids typically weigh between 2 and 4.01 kg at birth, and they can gain up to 12.9 kg [10–12]. Body weight increments and body condition score (BCS) are crucial components of farm management because they may be used to calculate the amount of muscle and fat that will develop over time. Animals use fat as an energy store to get them through high-stress situations, such as health problems or food deficiencies [13]. Animals with low BCS and body weight will lack the energy required to function properly, resulting in much-decreased performance [14]. The BCS is a subjective method for assessing a flock’s nutritional condition and may serve as a cue for goat breeders to boost.

Smallholders are starting to open more dairy goat farms in Malaysia because of the rising demand for animal products. However, this is insufficient to meet demand, and Malaysia must import goat products, mainly milk and meat, from other countries [2]. One of the breeds of dairy goats with the largest bodies is the Saanen goat, which may mature at a live weight of up to 55 kg and a height of up to 80 cm [3]. Saanen goats are also referred to as dual-purpose breeds because they can produce both milk and meat. Goat milk is well-liked in Malaysian society because of its medicinal benefits and religious importance [4,5]. Goat farming is one of the important industries in the agro-food subsector in Malaysia [6]. Goats provide a source of meat and milk for Malaysians’ nutritional requirements. Currently, 29.5% of registered livestock farms in west Malaysia are devoted to small ruminant populations [7]. Goat milk is essential to produce cheese and yogurt instead of milk and meat. Weaning young animals should therefore take the place of breeding stock whenever possible because doing so increases farm output and satisfies the objectives of the dairy sector, provided that the animal’s ability to live and grow unharmed is provided [8,9,15]. st their herd’s Goats with a good BCS and enough food, on the other hand, shouldn’t be too fat or too skinny [16].

The weaning stages in this study encompass pre-and post-weaning on a local Saanen dairy goat farm. On this farm, Saanen kids are frequently underweight, which causes the farmer great concern. Previous research has shown that gender can influence growth parameters because of the positive interaction between male gonadal cells and growth hormones, which are linked to growth performance [17]. According to Kari et al. [18], there are two key measures to evaluate goat kids’ productive efficiency, which are body size and weight. To boost goat production, these variables are essential. Farmers mostly keep track of disease outbreaks and parities that take place on the farm, with little data on kids were recorded. In addition, it is difficult to determine the BCS of pre-weaning kids because they are still in a growing stage. Since milk is the only food given to Saanen kids who are in the weaning period, it takes them longer to put on weight.

In contrast to other land-dwelling mammals, goat growth and development follow a similar pattern and are influenced by both external and internal climate influences. Saanen kids have varying live weights before and after weaning, which affects how they wean [19]. The growth curve of the Saanen children before and after weaning can be calculated at the conclusion of this study. On the Saanen goat kids’ growth, the gender influence will be determined. Additionally, this can assess the Saanen kids’ BCS and measure their growth rates. The Saanen kids’ body weight and increase in live weight over time are estimated using the growth curve [20] in both pre-and post-weaning situations. Additionally, the growth curve assists in the development of feeding schedules and farm management planning, including animal performance and overall output [21].

The study is most important to lessen and achieve non-biased effects on the BCS determined. Thus, it helps the farmers to enhance the quality of their production of Saanen kids on a local dairy goat farm. The results are important to enhance the production of Saanen kids to grow healthy. There are three objectives in this study: to measure the weekly live weight of male and female pre-and post-weaning Saanen kids; to evaluate the BCS of pre-and post-weaning Saanen kids; and to determine the growth curves between males and females of pre-and post-weaning Saanen kids on a dairy goat farm.

## Materials and Methods

### Ethics approval

The sampling methodology and experimental protocols employed in this work were authorized by the UniSZA Animal and Plant Research Ethics Committee (reference number: UAPREC/007/032).

### Description of study environment

The study was conducted on the local dairy farm, Besut District, Terengganu. The geographical location is between 5 38’N and 102˚28’E. The average annual temperature in Besut is 26.9°C. Besut has a rainy season in January, June, July, August, September, October, November, and December.

### Goat selection procedure

Pre-weaning Saanen kids aged 1 month and post-weaning kids aged 3 to 6 months were employed for this study. These animals were selected based on the date of birth recorded in animal management records. The pre-and post-weaning kids were placed in Saanen kids’ pens in an intensive housing system where they are limited to grazing. Post-weaning kids were fed twice per day, with pellets in the morning and fresh *Brachiaria humidicola* in the afternoon. Ten Saanen kids (*n* = 10), 5 males and 5 females pre-weaning, and 20 Saanen kids (*n* = 20), 10 males and 10 females post-weaning, were used in this study. The live weight of each of the pre-and post-weaning Saanen kids was taken every Tuesday morning until week 6.

### Determining Saanen kids’ body weights

Pre-weaning Saanen kids were marked on their heads, while post-weaning Saanen kids were marked on their backs. This allowed us to differentiate the samples from other Saanen kids using marking crayons and sex identified. There were 10 pre-weaning (5 males and 5 females) and there were 20 (10 males and 10 females) marked in the same pan. The kids were selected and restrained carefully before being weighed. When it came to weight, one end of the rope was tied to each kid’s shoulders, and the other end was tucked under their bellies. A weighing scale with a 100% sensitive balance scale was used to weigh the kids. The kids were weighed once weekly using the same weighing scale upon the mark. The live weight of the pre-and post-weaning Saanen kids was recorded. Thus, an analysis of the performance of growth was recorded. The statistical analysis was determined after weighing the pre-weaning and post-weaning kids.

### Statistical analysis

The effects of gender on the growth performances of pre- and post-weaning Saanen kids’ were analyzed using a two-sample *t*-test in Minitab Software (version 17.1, State College, PA). The *t*-test was done to present the average of live weight ± SD and average of BCS ± SD along with the level of significance *p* < 0.05.

## Results and Discussion

### Pre-weaning Saanen kids

**Figure 1.**
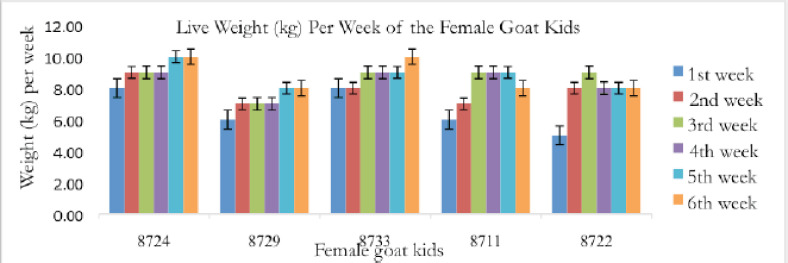
Live weight (kg) of the female goat kids across a 6-week sample period.

**Figure 2.**
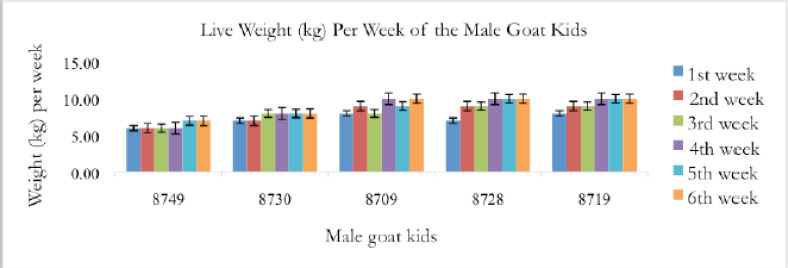
The male kids’ live weight (kg) was measured during a 6-week period.

For this study, 10 pre-weaning kids (5 males and 5 females) of the same age were used. The pre-weaning Saanen kids’ live weights and growth patterns were shown in Figures 1 and 2.

Table 1 shows that the result of an average live weight ± SD of the pre-weaning Saanen goat kids differed non-significantly (*p* > 0.05) over the weeks. Pre-weaning kids’ average live weights were highest at week 6, when they were 9.00 ± 1.41 and 8.80 ± 1.10 kg, respectively. As a result, pre-weaning kids’ body weight varies from the 1st to 6th week as their growth rate increases. The average live weight of females, however, remained the same in weeks 5 and 6. Overall, both males and females exhibit a consistent increase in live weight. It appears that both males and females had the same average live weight in weeks 3 and 4. The pre-weaning kids were in a stage of steady growth.

**Figure 3.**
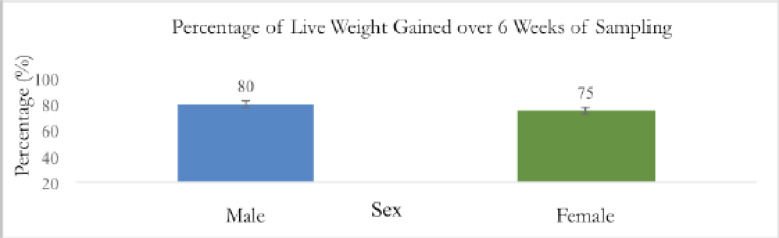
The percentage of live weight increased throughout the sampling period of 6 weeks.

Figure 3 shows the percentage (%) of live weight values gained over the 6-week sampling of pre-weaning Saanen kids. The graph demonstrates that male Saanen pre-weaning kids had a higher percentage (%) of live weight gained over a sampling period of 6 weeks. The weight gain of the two sexes does not, however, differ significantly.

**Table 1.** The average live weight (kg) of the male (*n =* 5) and female (*n =* 5) Saanen goat kids over six weeks of sampling (average live weight ± SD).

Study weeks	Average live weight ± SD	
	Male	Female
1 2 3 4 5 6	7.20 ± 0.84 7.80 ± 1.30 8.00 ± 1.22 8.40 ± 1.52 8.60 ± 1.14 9.00 ± 1.41	6.60 ± 0.60 7.80 ± 1.34 8.00 ± 0.71 8.40 ± 0.89 8.80 ± 0.84 8.80 ± 1.10

Values within a column show non-significant differences (*p* > 0.05).

**Figure 4.**
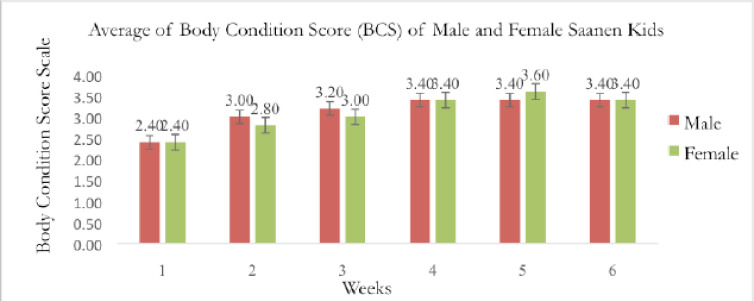
BCS of male and female pre-weaning Saanen kids.

Figure 4 shows the average BCS of pre-weaning Saanen kids of both sexes. Due to the graph’s consistent pattern, it can be observed that both male and female pre-weaning Saanen kids have normal BCS. Since they had just finished milking the colostrum a few weeks prior, both males and females began with the same average BCS. The BCS was therefore the same. The goats’ body size made it difficult to measure the fat cover because it was not uniform. A valid study was shown by the non-significance (*p* > 0.05).

Table 2 shows that the result of the average BCS ± SD of male and female pre-weaning Saanen goat kids differed non-significantly (*p* > 0.05) over the weeks. The male and female pre-weaning kids’ highest mean BCS values, 3.40 ± 0.89 and 3.60 ± 0.55, respectively, were discovered at week 6. Because they were in the growth phase, pre-weaning kids develop body fat quickly as they transition to post-weaning. When looking at the kids’ fat covers to see if they have a good BCS, it is important to consider the increase in muscle mass as the BCS goes up by weeks.

Pre-weaning kids’ live weight was revealed to be insignificant in a previous study [19] because they were still in the growing stage. The variations in kids’ live weight during the 1st month before weaning were possibly caused by the fact that lighter kids did not experience weaning shock as severely as heavier kids, whose growth had slowed after weaning. Most likely, the milk of the dams causes variations in the kids’ live weight. Kids who grew at different rates as a result of the impacts of dam milk may create a more homogeneous population after weaning due to normal feeding. Thus, it is explained in Figures 1 and 2 that in the 1st month of age, the live weight of the kids during the 1st week of study is slightly different from one another.

Portolano et al. [22] also reported that between the first 2 weeks of age and over 1.5 to 2 months of age, the dam’s age had a substantial effect on the pre-and post-weaning growth periods, but not on the weaning period (between 30 and 45 days old). Compared to goats over the age of 8 to 10 years, kids born from older goats were heavier at birth. However, according to Al-Shorepy et al. [23], the age of the dams at Kidd had no discernible effects on any features.

According to a previous study [17], the positive correlation between male gonadal cells and growth hormones, which are linked to growth performance, may explain how gender affects growth features. Male pre-weaning kids have consistently greater live weights than female pre-weaning kids (Table 1), which is consistent with the findings of other authors [12,24]. On the other hand, Ndlovu and Simela [25] found that between 90 and 180 days of age, a kid’s gender had no influence on their body weight or rate of growth. Hary and Schwartz’s [26] reports state that as animals get older, the weight gap between males and females rises. These benefits to male kids’ growth potential that were observed in this study are comparable to those that were reported for other goat breeds [23]. Male precocity may make clear why males are superior to females.

Live weight is one of the crucial criteria for animal selection [27]. Live weight is also one of the key elements in evaluating potential milk production as well as several other traits of farm animals, especially those with significant economic value. So, it is not surprising that farmers always pay a lot of attention to how much a goat kid weighs when it is still alive.

**Table 2.** The average BCS of the male (*n =* 5) and female (*n =* 5) Saanen goat kids over 6 weeks of sampling (average BCS ± SD).

Weeks	Average of BCS ± SD	
	Male	Female
1 2 3 4 5 6	2.40 ± 0.55 3.00 ± 1.00 3.20 ± 0.84 3.40 ± 0.89 3.40 ± 0.89 3.40 ± 0.89	2.40 ± 0.55 2.80 ± 0.84 3.00 ± 0.71 3.40 ± 0.89 3.60 ± 0.55 3.60 ± 0.55

Values within a column show non-significant differences (*p* > 0.05).

Due to their growth stage, pre-weaning kids’ BCS is challenging to assess. Thus, the results gathered most were BCS 2 and 3, as the pre-weaning kids have normal BCS and are undergoing normal growth performances. There was no paper reporting the BCS in pre-weaning kids because they were still in a growing phase. The BCS can, however, be used to find out if the milk the babies drank before they were weaned was enough for them.

### Post-weaning Saanen kids

**Figure 5.**
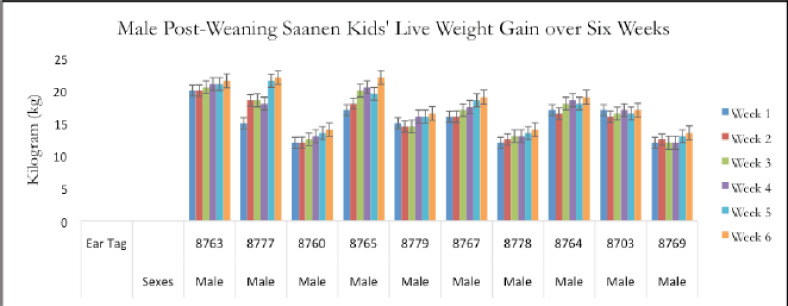
Live weight (kg) of the male kids over a sample period of 6 weeks.

**Figure 6.**
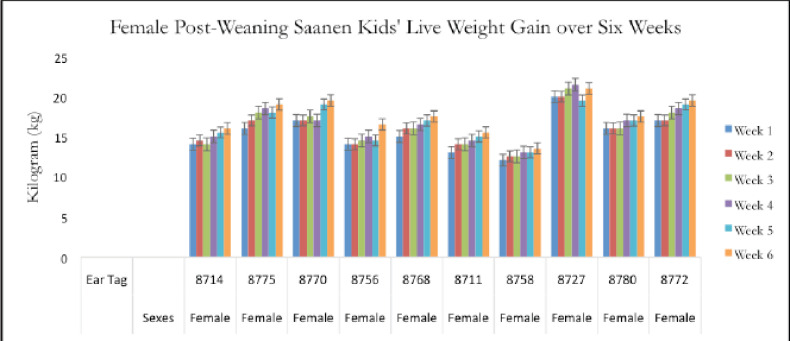
Live weight (kg) of the female kids across a 6-week sample period.

Twenty post-weaning Saanen kids aged 3–6 months old were employed for this study. The live weight values represent the respective growth rates of selected post-weaning Saanen kids at a local dairy goat farm (Figs. 5 and 6).

Table 3 shows that the result of an average live weight ± SD of the male post-weaning Saanen goat kids is non-significant (*p* > 0.05) over the weeks. At week 6, male and female post-weaning kids had the greatest average live weights, 17.85 ± 3.36 and 17.55 ± 2.25 kg, respectively (Table 3). As a result, there are variations in body weight in kids from the first to the 6th week post-weaning due to growth increases. As a result, from the 1st to 6th week after weaning, kids’ body weight varies because of their increased growth. The post-weaning kids were at a stage of stable growth.

**Figure 7.**
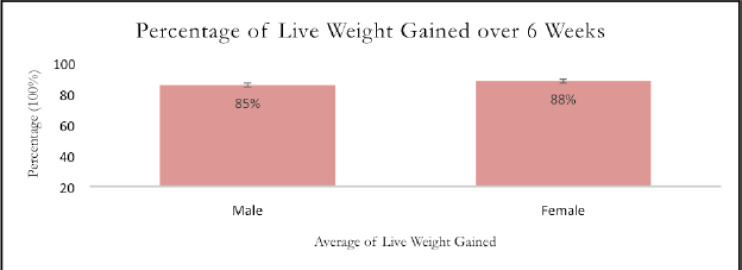
The percentage of live weight gained by post-weaning Saanen kids over 6 weeks.

The percentage of live weight values that post-weaning Saanen kids gained during the period of a 6-week sampling is shown in Figure 7. The proportion of females is 3% higher than the percentage of males. The graph demonstrates that female Saanen post-weaning kids have a higher percentage (%) of live weight gained over a sample period of 6 weeks. The weight growth of the two sexes does not, however, differ much.

**Table 3.** The average live weight (kg) of the male (*n =* 10) and female * n =* 10) Saanen goat kids over 6 weeks of sampling (average live weight ± SD).

Weeks	Live weight in kg (mean ± SD)	
	Male	Female
1	15.30 ± 2.67	15.40 ± 2.32
2	15.65 ± 2.75	15.80 ± 2.14
3	16.25 ± 3.10	16.15 ± 2.53
4	16.65 ± 3.14	16.65 ± 2.45
5	17.10 ± 3.12	16.75 ± 2.19
6	17.85 ± 3.36	17.55 ± 2.25
Total mean	16.47 ± 3.02	16.38 ± 2.31

Values within a column show non-significant differences (*p* > 0.05).

**Figure 8.**
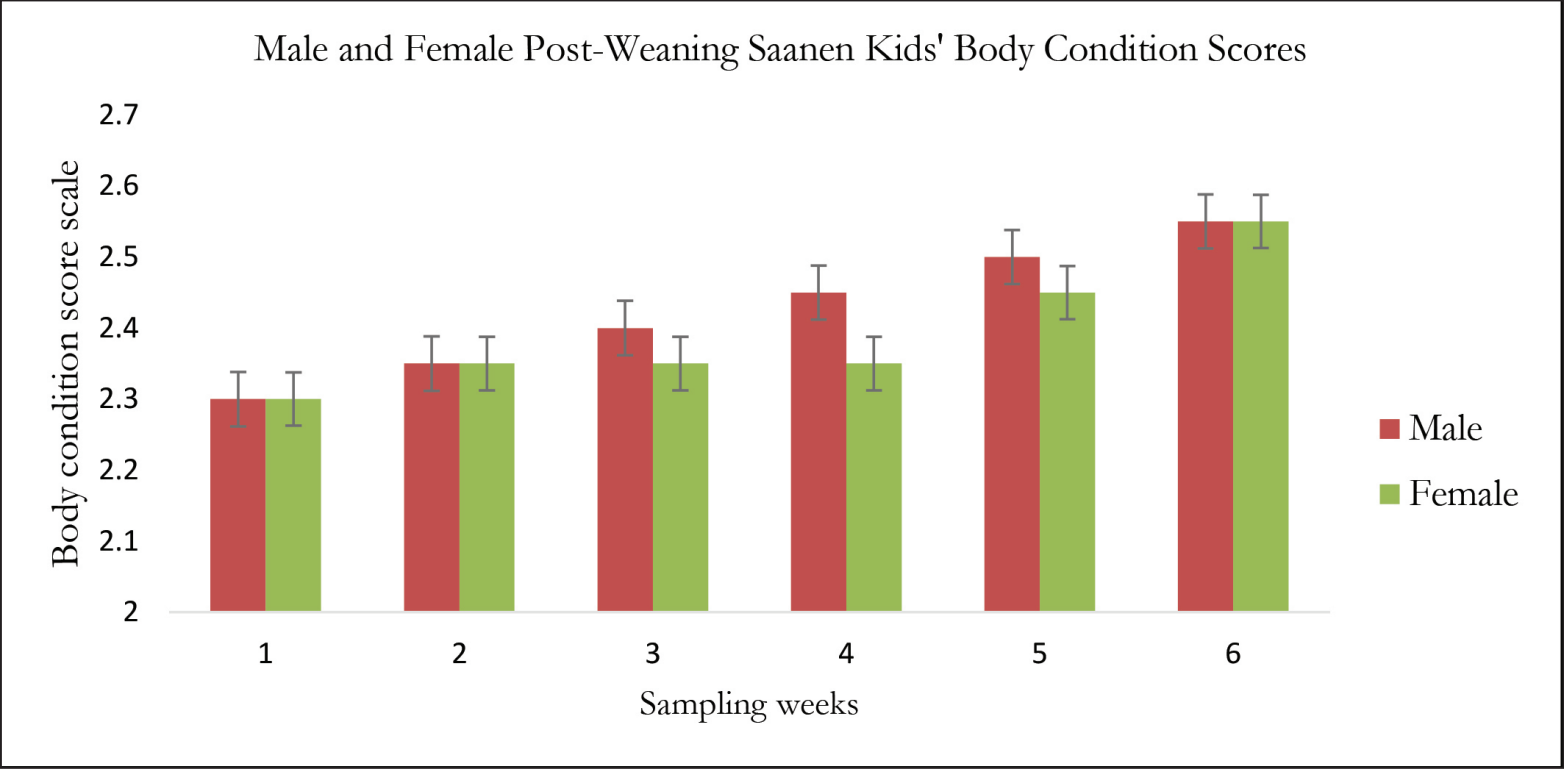
The average graph for BCS versus sexes post-weaning Saanen kids.

The average BCS for post-weaning Saanen kids across the sexes is shown in Figure 8. The graph demonstrates that both male and female post-weaning Saanen kids have typical BCS since it displays a consistent pattern. Saanen kids require time to adjust to their new surroundings. Both males and females begin with the same average BCS due to the same factor, which is stress from being separated from the mother. The BCS, therefore, remained consistent between weeks 1 and 2.

According to Table 4, there was no statistically significant difference in the average BCS ± SD of male and female post-weaning Saanen kids throughout the period of 6 weeks. At week 6, the post-weaning kids’ highest mean BCS values for males and females were 2.550 ± 0.438 and 2.550 ± 0.550, respectively (Table 4). Because they are in the growth phase, post-weaning kids develop body fat quickly as they get older. The Saanen kids are acquiring muscle and fat in their bodies, as seen by the BCS being increased by weeks.

**Table 4.** The average BCS of the male (*n =* 10) and female (*n =* 10) Saanen goat kids over 6 weeks of sampling (average BCS ± SD).

Weeks	BCS (mean ± SD)	
	Male	Female
1	2.300 ± 0.350	2.300 ± 0.350
2	2.350 ± 0.337	2.350 ± 0.337
3	2.400 ± 0.394	2.350 ± 0.337
4	2.450 ± 0.369	2.350 ± 0.337
5	2.500 ± 0.408	2.450 ± 0.438
6	2.550 ± 0.438	2.550 ± 0.550
Total mean	2.425 ± 0.383	2.392 ± 0.392

Values within a column show non-significant differences (*p* > 0.05).

According to the current findings, which are shown in Figures 5 and 6, the average live weight gain of post-weaning Saanen kids in 6 weeks is greater in males than in females. The data show the same finding as in the previous study [12] that there was no mortality in reared kids during the experiment. Following weaning, there were no significant differences in the live weight gained between male and female Saanen kids (*p* > 0.05).

For a 6-week sampling period, the female kids in Figure 7 had a higher proportion of live weight than the male kids. These results were in direct opposition to a prior study [12]. Males typically experience a higher percentage of live weight gain during this time. According to a recent study, male kids may be more resilient to the stress of weaning than females because females lose their benefits after weaning. This is because the kids raised on this farm are not completely in control of the environmental effects. Environmental impact is one of the factors that affect the growth rate of goat kids [28]. The temperature during the sample period was high and unsuitable for the post-weaning Saanen kids. Male kids were more active than female kids during the sampling period, which had an impact on the percentage of live weight gain.

According to Figure 8, both the male and female kids’ body weight increased, and there was no relationship between the kids’ BCS and live weight. This study confirms the findings of the earlier studies [29,30], which also found that changes in goats’ body weight did not always correspond to measurements of their physical condition. Body weight is affected by gut contents, which are entirely dependent on the feeding regimens of the goats. Hence, BCSs have been found to reflect body lipids than body weight [31,32] more precisely.

## Conclusion

The live weight and BCS of pre- and post-weaning Saanen kids were monitored and determined as part of this direct assessment study. At the age of 1 month, the average live weight of pre-weaning kids was determined to be inconsequential. The environment, farm management, and milk consumption from mothers all have an impact on the mean live weight. The pre-weaning Saanen kids’ growth would be impacted by the gender differences in live weight and BCS body composition. Comparison to the male post-weaning kids confirms the fact that the female Saanen kids have a somewhat larger average live weight growth over the period of 6 weeks. Overall, the live weight of the kids is not related to the BCS.

## List of Abbreviations

%, percentage; BCS, Body condition score; BCS ± SD, Body condition score ± standard deviation; live weight ± SD, Live weight ± standard deviation; UniSZA, Universiti Sultan Zainal Abidin.
